# Supplementary material for: Learning curve and surgical outcome of robotic assisted colorectal surgery with ERAS program
Source: Sci Rep. 2022 Nov 29;12:20566. doi: 10.1038/s41598-022-24665-w (PMC9709162; doi:10.1038/s41598-022-24665-w)
Supplement: Supplementary file 2 — Supplementary Information 2. [file 41598_2022_24665_MOESM2_ESM.zip › RAL Raw data-Ñ[▒K/141/Patho_ñw╝╨░O▒Kñσ.pdf]

臺中榮民總醫院 臺中市臺灣大道四段1650號 病理部活體組織切片 臨床醫學實驗及病理診斷中心 檢驗(查)報告

[redacted]

Pathologic diagnosis:
1. Rectum, robotic low anterior resection --- Status post polypectomy with edema and mild inflammation in the submucosa. No residual tumor is seen. The bilateral cut ends and perirectal lymph nodes (0/17) are also free of tumor.
2. Rectum, cut end, distal, resection --- Unremarkable.
3. IMA root, dissection --- Unremarkable.

Prognostic and predictive factor:
1. Tumor size: Not applicable.
2. Depth of invasion: No evidence of primary tumor.
3. Histologic grade: Not applicable.
4. Angiolymphatic invasion: Not identified.
5. Perineural invasion: Not identified.
6. Tumor deposits (discontinuous extramural extension): Not identified.
7. Macroscopic tumor perforation: Not identified.
8. Surgical Margins:
a) Proximal Margin: Uninvolved by invasive carcinoma.
b) Distal Margin: Uninvolved by invasive carcinoma.
c) Radial or Mesenteric Margin: Uninvolved by invasive carcinoma.
9. Lymph node status: Uninvolved (0/17).
10. Extranodal involvement: Not applicable.
11. Tumor regression grading S/P CCRT: No known presurgical therapy.
12. Type of polyp in which invasive carcinoma arose: Not identified.
13. Additional pathologic findings: Not identified.
14. Pathological TNM Stage: pTxN0 (According to the eighth edition, American Joint Committee on Cancer Staging Guidelines for Tumors).
15. TNM descriptors: Not applicable.

Gross description:
The specimen consists of 1) a segment of colon, 13.4 cm in length and 5.5 cm in proximal cut end circumference, 4.6 cm in distal circumference, and perirectal soft tissue. A stitch over an area with black dye, is noted, 4.5 cm to the distal cut end. On cross section, no tumor is found grossly. 2) IMA root, a piece of tan yellow soft tissue, 1.3x0.7x0.4 cm. 3) distal cut end, a piece of tan mucosa-covered soft tissue, 2x2x1.2 cm. Representative part for section: A) proximal cut end B) distal cut end C-D) colon mucosa on the stitch site E-F) colon mucosa close to the stitch G) specimen 2 H) specimen 3 J-L) perirectal lymph nodes.
#T-59600_2 #M-09413_2 0 1105 SP0000
#T-C4560_2 #M-09183_2 0 1105 000000

已電子簽章

警告：本單僅供醫師參考使用，不具任何申請效力！！
請病患妥為保管，若不慎遺失需自負個人資料外洩責任！
